# Association of cigarette smoking with cerebrospinal fluid biomarkers of insulin sensitivity and neurodegeneration

**DOI:** 10.1002/brb3.3432

**Published:** 2024-02-15

**Authors:** Fan Wang, Hui Li, Tiantian Kong, Ligang Shan, Jiajia Guo, Yan Wu, Xingguang Luo, Senthil Kumaran Satyanarayanan, Kuan‐Pin Su, Yanlong Liu

**Affiliations:** ^1^ Beijing Huilongguan Hospital Peking University Beijing China; ^2^ Department of Biomedical Engineering College of Future Technology Peking University Beijing China; ^3^ Xinjiang Key Laboratory of Neurological Disorder Research the Second Affiliated Hospital of Xinjiang Medical University Urumqi China; ^4^ Department of Anesthesiology the Second Affiliated Hospital of Xiamen Medical College Xiamen China; ^5^ Medical Section The Third Hospital of BaoGang Group Baotou China; ^6^ The Affiliated Hospital of Inner Mongolia Medical University Huhhot China; ^7^ Department of Psychiatry Yale University School of Medicine New Haven USA; ^8^ Department of Psychiatry & Mind‐Body Interface Laboratory (MBI‐Lab) China Medical University Hospital Taichung Taiwan; ^9^ College of Medicine China Medical University Taichung Taiwan; ^10^ An‐Nan Hospital China Medical University Tainan Taiwan; ^11^ Zhejiang Provincial Clinical Research Center for Mental Disorders, The Affiliated Wenzhou Kangning Hospital Wenzhou Medical University Wenzhou China

**Keywords:** amyloid‐β 42, cerebrospinal fluid, cigarette smoking, insulin, single nucleotide polymorphism

## Abstract

**Introduction:**

Cigarette smoking increases both the risk for insulin resistance and amyloid‐β (Aβ) aggregation, and impaired brain insulin/insulin‐like growth factor 1 (IGF1) signaling might increase risk factors for Alzheimer's disease (AD). We aimed to investigate the association among cerebrospinal fluid (CSF) insulin sensitivity/IGF1, glucose/lactate, and Aβ42 and further explore whether insulin sensitivity contributed to the risk for AD in active smokers.

**Methods:**

In this cross‐sectional study, levels of insulin, IGF1, and lactate/glucose of 75 active smokers and 78 nonsmokers in CSF were measured. Three polymorphisms regulating IGF1 were genotyped. Analysis of variance was used to compare differences of variables between groups. Partial correlation was performed to test the relationship between CSF biomarkers and smoking status. General linear models were applied to test the interaction of the effect of single nucleotide polymorphisms and cigarette smoking on CSF IGF1 levels.

**Results:**

In the CSF from active smokers, IGF1 and lactate levels were significantly lower (*p* = .016 and *p =* .010, respectively), whereas Aβ42 (derived from our earlier research) and insulin levels were significantly higher (*p* < .001 and *p* = .022, respectively) as compared to the CSF from nonsmokers. The AG + GG genotype of rs6218 in active smokers had a significant effect on lower CSF IGF1 levels (*p* = .004) and lower CSF insulin levels in nonsmokers (*p* = .016).

**Conclusions:**

Cigarette smoking as the “at‐risk” factor for AD might be due to lower cerebral insulin sensitivity in CSF, and the subjects with rs6218G allele seem to be more susceptible to the neurodegenerative risks for cigarette smoking.

## Graphical abstract

A flowchart of the selection of participants

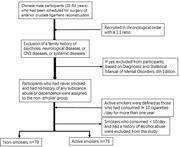

## 1 INTRODUCTION

Brain insulin resistance (IR) is a risk factor for Alzheimer's disease (AD) (Talbot et al., 2012). Compared with nonsmokers, active smokers had a significantly higher homeostatic model assessment for IR index (Seet et al., 2012). Cigarette smoking increases the risk for IR and amyloid‐β (Aβ) aggregation (Moreno‐Gonzalez et al., 2013; Xie et al., 2009), but their association with AD remains elusive.

The core biomarker for the specific diagnosis of AD, the 42 amino acid Aβ isoform Aβ42, is associated with IR, promotes plaque formation, and plays a key role in the pathogenesis of AD (Teunissen et al., 2018), and it has been demonstrated that levels of cerebrospinal fluid (CSF) Aβ42 increased during the very early phase of cerebral Aβ deposition in mouse models (Maia et al., 2015), whereas the reduced insulin sensitivity impairs the role of insulin in inhibition of Aβ42 aggregation and Aβ42‐induced neuron damaged prevention (Long et al., 2019). Cumulative data have reinforced that the brain is an insulin‐sensitive organ (Kullmann et al., 2016). It has been found that postmortem brain tissue from patients with AD and animal models manifests characteristics of IR even in the absence of systemic IR (Mullins et al., 2017). Hyperinsulinemia reflected in CSF chronically elevates insulin and renders neurons resistant to insulin (Talbot, 2014). Insulin works on astrocytes to control the neuronal response to glucose availability (Garcia‐Caceres et al., 2016), and a rise in insulin attenuating blood glucose is well known as another characteristic of IR (Cavaghan et al., 2000). Insulin‐like growth factor 1 (IGF1) has an almost 50% amino acid sequence homology with insulin and elicits nearly the same hypoglycemic response as insulin (Boulware et al., 1994). IGF1 impacts insulin's ability to maintain normal carbohydrate homeostasis, and the treatment of IGF1‐deficient mice with exogenous IGF1 inhibits insulin secretion and improves insulin sensitivity (Yakar et al., 2004).

Further, impaired insulin/IGF1 signaling in the brain not only has been linked to increased risk for premature cognitive decline, dementia, and AD but also increases Aβ42 level (Long et al., 2019), causing hypometabolism of neurons (Sivanesan et al., 2018). AD patients show global reductions in brain glucose metabolism relative to normal healthy controls (Waldron et al., 2015), whereas glucose can enhance the clearance of Aβ42 from the brain and stimulate brain energy metabolism (Jauch‐Chara et al., 2012). At this time, lactate is generally used preferentially over glucose by neurons to resist Aβ42 toxicity for nerve cells to enable long‐term memory (Jauch‐Chara et al., 2012). Nevertheless, how glucose and lactate are associated with AD when brain IR occurs remains unknown. Therefore, although brain IR can manifest as an impaired ability to regulate metabolism (Arnold et al., 2018), the relationship between cerebral insulin sensitivity and glycometabolism in AD needs to be further addressed.

It is worth noting that cigarette smoking not only exacerbates Aβ aggregation (Moreno‐Gonzalez et al., 2013) and directly increases the risk for IR (Xie et al., 2009) but also negatively regulates IGF1 (Erlandsson et al., 2016). Although the roles of insulin/IGF1 in cerebral energy metabolism have been reported before, the metabolic effects of brain insulin, independently from plasma insulin levels, are very important (Arnold et al., 2018). Additionally, cigarette smoking can affect glucose/lactate metabolism (Huie et al., 1996). This study was conducted to investigate CSF levels of insulin/IGF1, glucose/lactate, and Aβ42 between nonsmokers and active smokers to explore whether the changes of cerebral insulin sensitivity contribute to the AD risk in active smokers.

Additionally, as the variants of promoter and 3′ untranslated region (UTR) could regulate their target genes mRNA expression and downregulate translation degree (Mao et al., 2017). Consequently, the three single nucleotide polymorphisms (SNPs) located on the promoter and the 3′ UTR of IGF1 gene (Aoi et al., 2010; Xie et al., 2008; Zhao et al., 2007), rs5742612, rs6218, and rs6214, were genotyped to further explore the possible effect of specific alleles on cerebral insulin sensitivity in active smokers.

## 2 METHODS

### 2.1 Study design

This cross‐sectional study was conducted following the Strengthening the Reporting of Observational Studies in Epidemiology guideline. This study was approved by the institutional review board of Inner Mongolian Medical University and was performed in accordance with the Declaration of Helsinki. Written informed consent was obtained from all participants, and no financial compensation was provided to the participants. The cases were defined as individuals who actively smoke, and controls were matched individuals who do not smoke.

### 2.2 Participants

Due to few female smokers in China (Ma et al., 2017), no smoking females were recruited in the present study. A total of 153 Chinese male participants (18–64 years) who had been scheduled for surgery of anterior cruciate ligament reconstruction were recruited from September 2014 to January 2016 (before Chinese version of the Diagnostic and Statistical Manual of Mental Disorders, 5th published). Of these, 75 were active smokers, whereas 78 were nonsmokers. Sociodemographic data, including age, years of education, and body mass index (BMI), were collected, and exclusion criteria were as follows: (1) a family history of psychosis or neurological diseases, or central nervous system (CNS) diseases determined by mini‐international neuropsychiatric interview, or (2) systemic diseases based on the medical history and admitting diagnosis.

Participants who had never smoked and had no history of any substance abuse or dependence were assigned to the nonsmoker group. Daily smoking was subgrouped into heavy smoking (at least 20 cigarettes daily) and moderate smoking (10–20 cigarettes daily) (Willi et al., 2007). Exposure to high doses of nicotine shows impairment in cognitive performance (Bentley et al., 2011). Consequently, active smokers were defined as those who had consumed half a pack of cigarettes (half pack = 10 cigarettes) or more per day for more than 1 year. Smokers who consumed fewer than 10 cigarettes per day were excluded from this study. According to the Diagnostic and Statistical Manual of Mental Disorders, 4th Edition, all participants had neither history of alcohol abuse nor psychiatric disorders.

Given that genetics analysis in the present study, we kept the geographic homogeneity of the participants (Manica et al., 2005).

### 2.3 Assessments and biological sample collection

Smoking‐related habit variables were obtained from active smokers, including age at smoking onset, years of cigarette smoking, the average daily number of cigarettes smoked, and the maximum daily number of cigarettes smoked.

The data of the routine tests, including high‐density lipoprotein, low‐density lipoprotein, alanine aminotransferase, cholesterol, triglyceride, gamma‐glutamyl transferase, aspartate aminotransferase, and the collection of CSF samples, were the same as the literature (Li et al., 2020).

### 2.4 Laboratory tests and genotyping

CSF analyses were performed to quantify levels of glucose and lactate using spectrophotometric kits (Nanjing Jiancheng Bioengineering Institute, Nanjing, China), Aβ42 using enzyme‐linked immunosorbent assay kit (Cloud Clone Corp.), and insulin and IGF1 (DIAsource ImmunoAssays S.A.) using radioimmunoassay kits.

Genomic DNA was extracted from white blood cells as same as the literature (Yang et al., 2017). Three loci were genotyped using the Sequenom MassARRAY platform (BioMiao Biological Technology). The primer sequences for rs5742612, rs6218, and rs6214 are listed in Table S1. The polymerase chain reaction (PCR) condition was set as shown: 95°C for 5 min, and (95°C for 20 s, 56°C for 30 s, and 72°C for 1 min) x45 cycles and 72°C for 3 min, then held at 4°C.

All experimental procedures followed the manufacturer's instructions, and the data analyst was blinded to the clinical data.

### 2.5 Statistical analysis

The homogeneity of variance was performed using Levene's test. Multicollinearity was calculated using stepwise multiple regression among covariates estimated via tolerance and variance inflation factor (VIF) as the cut‐off recommended thresholds for tolerance <0.1 and VIF >10 (Kim, 2019).

Polymorphisms were calculated using the SHEsis platform (Shi & He, 2005), including Hardy–Weinberg equilibrium and association analysis. Analysis of variance (ANOVA) and analysis of covariance (ANCOVA) were used to compare differences of variables between groups. Partial correlation analysis was performed to test the relationship between CSF biomarkers and smoking status, adjusting for the covariates of age, education, and BMI.

In addition, general linear models (GLM) were applied to test the significance of the interaction of the effect of SNPs and cigarette smoking on CSF IGF1 levels. The GLM model was constructed for an illustration of included dependency relationships: The model contains main effects of cigarette smoking and SNPs plus interactions of cigarette smoking and SNPs. Age, education, and BMI were included as cofactors in all models. The model was compared and tested using an *F*‐statistic.

Figure was created using GraphPad Prism version 8 (GraphPad Software Inc.) The statistical analyses were performed using IBM SPSS Statistics for Windows, Version 22.0 (IBM Corp.). Significant association of multiple comparisons for polymorphisms on GLM was applied using the Bonferroni‐corrected critical *p*‐value that sets the significance cut‐off at *α*/*n*. All tests were two‐sided and the statistical significance was defined at *p* < .05.

## 3 RESULTS

### 3.1 Demographic and clinical characteristics

The homogeneity of variance using Levene's test was performed for sociodemographic and clinical variables (all *p* > .05), except diastolic pressure. Consequently, ANOVA was used presented in Table 1.

**TABLE 1:** The differences of demographic and clinical characteristics between nonsmokers and active smokers.

Variables	Nonsmokers (*n* = 78)	Active smokers (*n* = 75)	*F*	*p*
Age (years)	31.97 ± 8.90	33.17 ± 10.16	.61	.44
Education (years)	13.12 ± 2.33	12.27 ± 2.81	4.00	.047*
BMI (Kg/m^2^)	24.98 ± 3.71	26.20 ± 3.53	4.32	.039*
Systolic pressure (mm/Hg)	130.60 ± 12.77	128.95 ± 13.05	.63	.43
Diastolic pressure (mm/Hg)	75.83 ± 8.81	77.32 ± 11.59	.80	.37
Glucose (mmol/L)	5.19 ± 0.44	5.27 ± 0.52	1.27	.26
High‐density lipoprotein (mM/L)	1.22 ± 0.23	1.22 ± 0.26	.16	.69
Low‐density lipoprotein (mM/L)	2.69 ± 0.55	2.65 ± 0.64	.10	.75
Alanine aminotransferase (U/L)	33.09 ± 24.82	31.93 ± 20.97	.10	.76
Cholesterol (mM/L)	4.76 ± 0.72	4.80 ± 0.86	.11	.75
Triglyceride (mM/L)	1.88 ± 0.89	1.88 ± 1.32	.00	.99
Gamma‐glutamyl transferase (U/L)	43.95 ± 33.59	46.80 ± 44.60	.20	.66
Aspartate aminotransferase (U/L)	22.50 ± 10.23	21.11 ± 7.44	.92	.34

*Note*: All data were reported as mean ± standard deviation using ANOVA.

Abbreviations: ANOVA, analysis of covariance; BMI, body mass index.

*p < .05.

Compared to nonsmokers, active smokers had lower education and greater BMI (13.12 ± 2.33 vs. 12.27 ± 2.81, *p* = .047 and 26.20 ± 3.53 vs. 24.98 ± 3.71, *p* = .039), whereas no difference was observed in other sociodemographic and clinical characteristics between two groups (Table 1).

### 3.2 Differences and correlations analysis of CSF biomarkers

**FIGURE 1:**
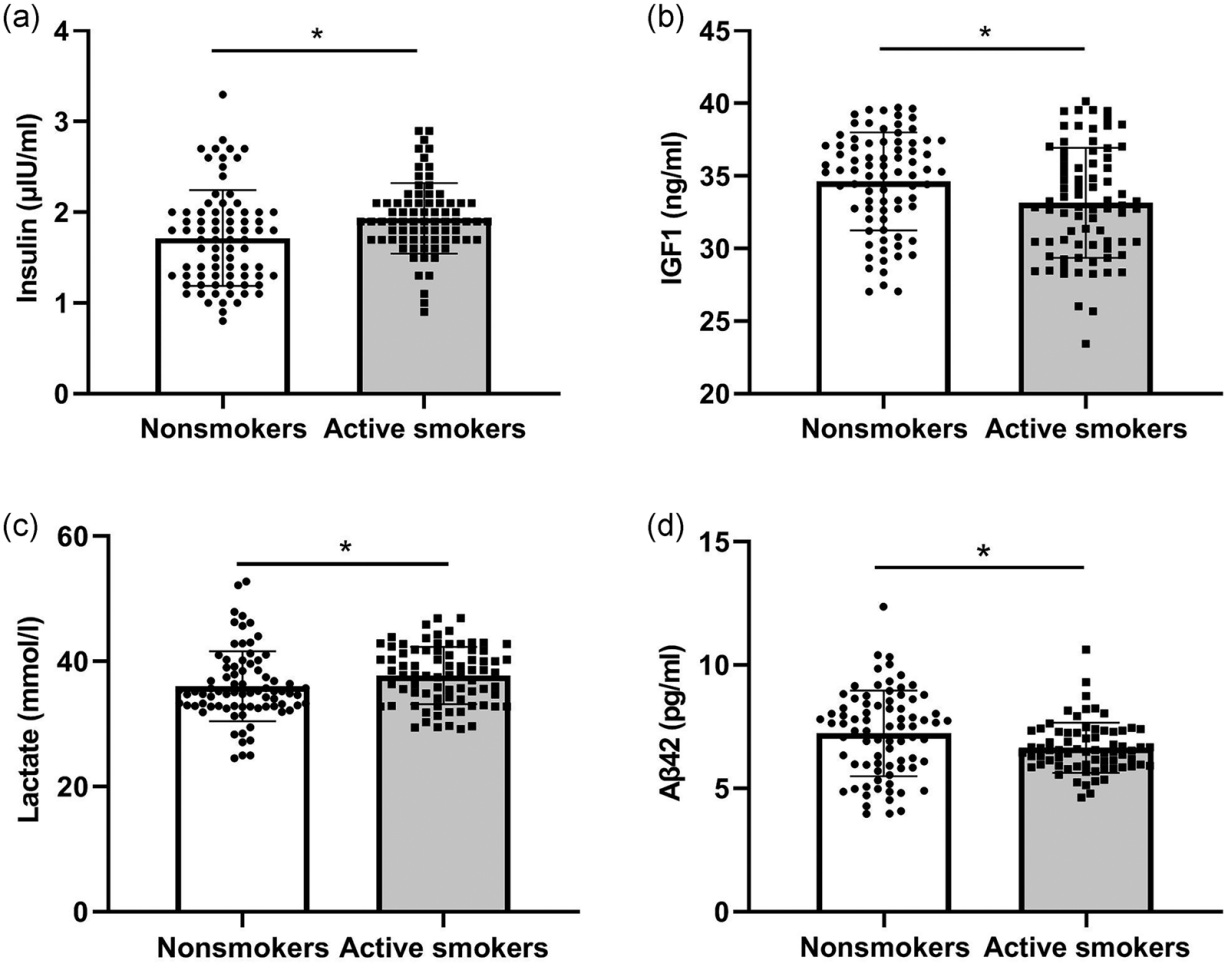
The differences of biomarkers in cerebrospinal fluid (CSF) between nonsmokers (*n* = 78) and active smokers (*n* = 75): (A) the differences of CSF insulin levels; (B) the differences of CSF insulin growth factor (IGF) 1 levels; (C) the differences of CSF lactate levels; (D) the differences of CSF amyloid‐β 42levels. *p < .05.

The homoscedasticity of residuals of four CSF biomarkers was also verified by Levene's test, and the results showed that the residuals of insulin and lactate were not contained in equal distribution (all *p* < .05). Stepwise multiple regression analyses of four CSF biomarkers—year, education, and BMI—showed that no variable was removed from models (all tolerance >0.9 and VIF <2). Therefore, ANCOVA was used to compare differences of four CSF biomarkers between two groups in Table 2. With age, education, and BMI as covariates, in nonsmokers, CSF levels of IGF1 and lactate were significantly higher (*p* = .016 and *p =* .010). In comparison, CSF levels of Aβ42 (this data were partly derived from our earlier research with 75 active smokers and 78 nonsmokers (Liu et al., 2020) and insulin were significantly lower (*p* < .001 and *p* = .022). The difference in glucose levels was not statistically significant between the two groups (*p* > .05) (Table 2 and Figure 1). But since collinearity of age with age at smoking onset and years of cigarette smoking, respectively, (both tolerance <0.1 and VIF >30), age was removed from models in active smokers. Therefore, partial correlation analysis was performed to test the correlation between CSF biomarkers and smoking‐related variables, adjusted for years of education, BMI, and other smoking‐related variables in active smokers, and there was no correlation (all *p* > .05).

**TABLE 2:** The differences of cerebrospinal fluid (CSF) biomarkers levels between nonsmokers and active smokers.

CSF biomarkers	Nonsmokers (*n* = 78)	Active smokers (*n* = 75)	Mean differences	95% CI	*F*	*p*
Insulin (μIU/mL)	1.71 ± 0.53	1.93 ± 0.38	−.18	−.34, −.03	5.34	.022*
IGF1 (ng/mL)	34.71 ± 3.34	33.18 ± 3.80	1.46	.27, 2.65	5.90	.016*
Lactate (mmol/L)	7.22 ± 1.75	6.69 ± 1.01	.66	.16, 1.16	6.92	.010*
Glucose (mmol/L)	3.03 ± 0.33	3.02 ± 0.36	−.01	−.13, .10	0.04	.83
Aβ42 (pg/mL)	36.56 ± 26.85	51.66 ± 17.54	−15.99	−24.03, −7.94	15.46	<.000*

*Note*: ANCOVA was used to calculate the differences of levels between two groups with age, education, and BMI as covariates.

Abbreviations: ANCOVA, analysis of covariance; Aβ42, amyloid‐β 42; BMI, body mass index.; CI, confidence interval; IGF1, insulin growth factor 1.

*p < .05.

### 3.3 Genetics analysis

There was no deviation from Hardy–Weinberg equilibrium of three loci (all *p* > .05). There were no significant differences in the distributions of alleles and genotypes of three loci between the two groups (all *p* > .05).

**FIGURE 2:**
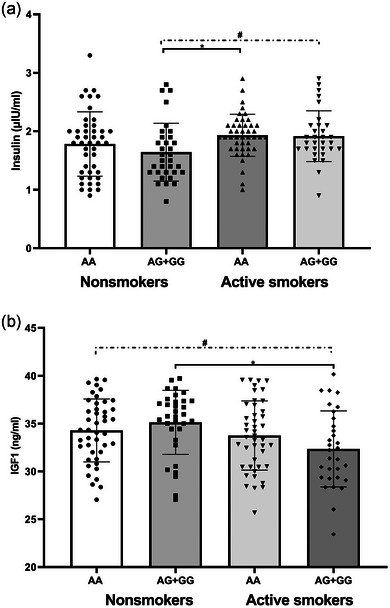
The differences of cerebrospinal fluid (CSF) insulin (A) and like growth factor 1 (IGF1) (B) levels of three genotypes of rs6218 between two groups; **^#^** suggestively significant, *p < .05.

To test for possible interactions between cigarette smoking and genotypic effects on phenotype, the GLM analyses of CSF IGF1 level were performed with age, education, and BMI as covariates. The GLM analysis in the data revealed a strong interaction for IGF1 level between rs6218 and cigarette smoking, and CSF IGF1 levels of the AG + GG genotype carriers in active smokers were significantly lower compared to the AG + GG genotype in nonsmokers (*p* = .004). In consideration of the possible role of IGF1 in insulin sensitivity, the GLM analysis was also calculated to test the interaction between the three genotypes and cigarette smoking on insulin level, adjusted for age, years of education, and BMI, and CSF insulin levels of the AG + GG genotype carriers in nonsmokers were significantly lower compared to the AA genotype in active smokers (*p* = .016). These interactions remained significant or suggestively significant after corrections for multiple testing (Table 3 and Figure 2).

**TABLE 3:** The interaction of rs6218 and cigarette smoking on cerebrospinal fluid (CSF) insulin and like growth factor 1 (IGF1) level.

Locus	CSF biomarkers	Simple effect (GLM)		Mean differences	*p*	*p_c_*
		Nonsmokers AA (*n* = 45); AG + GG (*n* = 33)	Active smokers AA (*n* = 44); AG + GG (*n* = 31)			
rs6218	Insulin (μIU/mL)	1.63 ± 0.08 _(AG + GG)_	1.92 ± 0.07 _(AA)_	−0.30	.004*	.016*
		1.63 ± 0.08 _(AG + GG)_	1.91 ± 0.08 _(AG + GG)_	−0.28	.014*	.056
	IGF1 (ng/mL)	34.21 ± 0.54 _(AA)_	32.36 ± 0.63 _(AG + GG)_	2.08	.013*	.052
		35.13 ± 0.61 _(AG + GG)_	32.36 ± 0.63 _(AG + GG)_	2.78	.001*	.004*

*Note*: GLM was used to calculate the differences of levels between four groups with age, education, and BMI as covariates. Simple effect was calculated using GLM. Data were reported as mean ± standard deviation. *p*_c_ was corrected using the Bonferroni correction.

Abbreviation: GLM, general linear models.

*p < .05.

There were no differences in CSF levels of glucose, lactate, and Aβ42 between the AA genotypes and the AG + GG genotypes in two groups, adjusted for age, years of education, and BMI, respectively (all *p* > .05).

## 4 DISCUSSION

We demonstrated an association of cigarette smoking with biomarkers of cerebral insulin sensitivity and neurodegeneration using CSF. The primary finding in the present study revealed that the association of cigarette smoking with low cerebral insulin sensitivity was indeed associated with the “at‐risk” factors of AD, as shown by higher levels of insulin and Aβ42 and lower levels of IGF1 and lactate in CSF of active smokers. No obvious changes in CSF glucose levels of active smokers’ further support the notion that decreasing cerebral insulin sensitivity was indeed associated with the at‐risk of AD in active smokers.

Chronic peripheral insulin elevations and insulin activity reduction means IR (Wilcox, 2005). Clinical studies have corroborated that patients with AD are more likely than healthy older adults to have reduced insulin sensitivity (Watson & Craft, 2004). Insulin and IGF1 modulate neuronal growth, survival, metabolism, synapse formation, and plasticity, and insulin, IGF1 polypeptides, and their receptors are expressed in the brain, most abundantly in regions that are most vulnerable to AD neurodegeneration (de la Monte & Wands, 2005). Insulin inhibits Aβ42 aggregation and prevents Aβ42‐induced membrane disruption (Long et al., 2019). In the mouse model of AD, it has been discovered that metformin, widely used to treat type 2 diabetes, enhances insulin sensitivity (Gupta et al., 2011), improves memory, and decreases concentrations of Aβ42. Another 14 population‐based cohort studies involving 396,332 participants were identified that metformin exposure was significantly associated with reduced risk of all subtypes of dementias, especially those who long‐term received metformin therapy AD patients (Ji et al., 2022). It further activates microglia, accompanied by improved brain insulin signaling, suggesting that the interaction of insulin and Aβ42 indeed exists in AD. Cerebral IR that results in increased Aβ production (De Felice, 2013) is increasingly recognized as an important factor of AD pathogenesis (Ng et al., 2016). In addition, animal models showed that reduced IGF1 led to hyperinsulinemia and abnormal glucose clearance (Sjogren et al., 2001). Moreover, intracerebroventricular injection of IGF1 improves glucose tolerance and insulin sensitivity in mice (Hong et al., 2017), which further enhanced the relationship between AD and cerebral insulin sensitivity.

It has been found that the decreases in cerebral lactate content may lead to the blockage of lactate transport from the glia to neurons, resulting in neuronal lactate deficit in the mouse brain, and the brain of AD patients remains in a state of cerebral hypometabolism (Zhang et al., 2018), and the hallmarks of impaired insulin sensitivity are decreased insulin‐stimulated glucose uptake into the insulin‐target tissues as an inadequate response by insulin‐target tissues to the physiologic effects of circulating insulin, which also means the decrease of blood glucose metabolism, the same as IR (Schenk et al., 2008). The phenomenon has been observed both in humans and animals that the spatial distribution of glycolysis in the brains of AD patients strongly correlates with Aβ42 deposition (Vlassenko et al., 2010), and the reduced utilization of glucose in rat brain elicits 250% of Aβ42 peptide production increase. In addition, the CSF glucose level is influenced by the serum glucose level. Fluctuations in blood glucose parallel changes in the CSF glucose (Nigrovic et al., 2012). The previous study found a dose‐response relationship between the number of cigarettes smoked per day and the degree of IR. Furthermore, an acute impairment in glucose tolerance and increased IR was observed in both nonsmokers and smokers following the consumption of three cigarettes (Artese et al., 2019), and cigarette smoke exposures also impair brain insulin/IGF signaling in mice (Deochand et al., 2016), and the blood glucose of participants in the present study was not abnormal. Therefore, in our results, accompanying the rise of insulin, no obvious changes in CSF glucose levels with active smokers indicated the existence of the changed insulin sensitivity and the risk for IR and higher levels of Aβ42. Moreover, as lactate as the production of glycolysis and is preferentially used over glucose for the sake of resistance against Aβ42 toxicity for nerve cells (Zhang et al., 2018), lower CSF lactate levels in active smokers in our results suggested a possible self‐ability/response of human bodies to resist AD risk.

Additionally, it is interesting to note that chronic cigarette smoke exposure not only could induce Aβ aggregation and IR that would be improved after its cessation (Moreno‐Gonzalez et al., 2013) but also negatively regulate IGF1 levels (Erlandsson et al., 2016). Despite no direct and pathogenetic evidence of cigarette smoking resulting in brain/periphery IR, previous studies have confirmed that serum insulin concentrations are higher in smokers compared to nonsmokers (Frati et al., 1996), and skeletal muscle IR in active smokers is associated with increased mammalian target of rapamycin activity and insulin receptor substrate‐1 Ser636 phosphorylation by nicotine. Similarly, maternal cigarette smoking downregulates of IGF1 signaling in fetal growth in a concentration‐dependent manner (Deng et al., 2013), which together suggests cigarette smoking is indeed able to result in a change of insulin sensitivity. Decreased CSF Aβ42 levels have been consistently found in the CSF of AD patients (Blennow et al., 2010). Postmortem studies have demonstrated inverse correlations between CSF Aβ42 and neuritic plaque burden suggesting that low levels of Aβ42 in CSF are caused by its deposition in the brain parenchyma (Strozyk et al., 2003). In addition, high positron emission tomography (PET) Aβ plaque deposition and low CSF Aβ42 are correlated (Fagan et al., 2006; Koivunen et al., 2008; Strozyk et al., 2003), as well one research finds CSF Aβ42 as a more sensitive marker of very early disease and PET Aβ deposition as a more sensitive marker of disease progression and downstream pathology (Mattsson et al., 2015). Therefore, higher CSF Aβ42 levels in our smokers were found. Given that cigarette smoking is considered as a risk factor of AD, induced IR and promoted Aβ aggregation have been reported already, and our results showed lower lactate and IGF1 levels and higher insulin and Aβ42 levels in CSF of active smokers, which suggested the association of cigarette smoking with low levels of brain energy metabolism and insulin sensitivity and the at‐risk biomarker of AD.

The genetic findings demonstrated that CSF IGF1 levels of the rs6218 AG + GG genotype carriers of active smokers were significantly lower, while there was a trend toward higher CSF insulin levels compared to the AG + GG genotype carriers of nonsmokers, suggesting that not only does cigarette smoking impacts IGF1 levels, but also the effect of the G allele on IGF1 levels is more susceptible to cigarette smoking than the A allele, as no differences of IGF1 levels of the A allele carriers were observed between the two groups (Figure 2A,B). Rs6218 was located in the 3′ UTR of the IGF1 gene (Aoi et al., 2010), and the G allele can effectively intervene in the binding of microRNA‐603 to the 3′ UTR region of IGF1, further increasing the transcription of IGF1 (Jiang et al., 2015). The previous study has reported that IGF1‐deficient mice with exogenous IGF1 inhibited the secretion of insulin and improved insulin sensitivity (Yakar et al., 2004), suggesting that the G allele might be protective against decreased insulin sensitivity. But smoking showed a negative regulation to serum IGF1 levels (Erlandsson et al., 2016), while in our results, CSF IGF1 levels of the rs6218 AG + GG genotype carriers of active smokers were significantly lower, suggesting that the G allele is more susceptible to the neurodegenerative risks for cigarette smoking. In addition, the A allele cannot intervene in the binding of microRNA‐603 to the 3′ UTR region. Consequently, our result also showed that the AA genotype carriers of active smokers had higher insulin levels than the AG + GG genotype carriers of nonsmokers, which implied the A allele carriers of active smokers may affect the interaction between smoking and insulin sensitivity (Figure 2B).

Cigarette smoking can have a protective effect against another neurodegenerative disorder, Parkinson's disease, as well previous epidemiological studies have explored the relationship between cigarette smoking and AD reaching contradictory conclusions (Piao et al., 2009). However, not only the functional connectivity of the striatum in healthy smokers was found to be negatively correlated with the severity of nicotine addiction, but also quitting smoking in the prodromal stage of AD may have the potential to prevent disease progression (Qiu et al., 2022). The present study has provided a theoretical basis for future preventive interventions for AD tailored to men.

There are some limitations to the present study. First, the insulin increase in the CSF cannot directly reflect the reduced brain insulin sensitivity; nevertheless, it represents biochemical changes in the brain compared to the peripheral phenomenon. Second, participants recruited in this study were presented with anterior cruciate ligament injuries instead of healthy populations, which might be a confounder when interpreting the results. Third, although only male smokers were recruited in the present study, the bias of gender differences of AD (Mazure & Swendsen, 2016) and the various effects of estrogen on reducing Aβ levels (Tamagno & Guglielmotto, 2022) were avoided.

## 5 CONCLUSION

Cigarette smoking was associated with the “at‐risk” biomarkers for AD as shown by the higher Aβ42 levels, excessive insulin, and lower IGF1 and lactate levels found in CSF of active smokers, and the subjects with rs6218G allele seem to be more susceptible to the neurodegenerative risks for cigarette smoking. These results broaden our understanding of the connection between cigarette smoking and neurodegenerative disorders.

## AUTHOR CONTRIBUTIONS

**Fan Wang**: Conceptualization; investigation; supervision; funding acquisition; writing—original draft; project administration; validation. **Hui Li**: Software; writing—original draft; data curation; formal analysis. **Tiantian Kong**: Writing—original draft; formal analysis. **Ligang Shan**: Resources; funding acquisition. **Jiajia Guo**: Resources. **Yan Wu**: Resources; funding acquisition. **Xingguang Luo**: Writing—review and editing. **Senthil Kumaran Satyanarayanan**: Writing—review and editing. **Kuan‐Pin Su**: Writing—review and editing; conceptualization; funding acquisition. **Yanlong Liu**: Conceptualization; validation; supervision.

## CONFLICT OF INTEREST STATEMENT

The authors report no financial interests or potential conflicts of interest.

### PEER REVIEW

The peer review history for this article is available at https://publons.com/publon/10.1002/brb3.3432.

## Supporting information

Table S1 The primers of three loci located on IGF1.

## Data Availability

The raw data supporting the conclusions of this article will be made available by the authors without undue reservation.
