# Supplementary material for: Association of cigarette smoking with cerebrospinal fluid biomarkers of insulin sensitivity and neurodegeneration
Source: Brain Behav. 2024 Feb 15;14(2):e3432. doi: 10.1002/brb3.3432 (PMC10869886; doi:10.1002/brb3.3432)
Supplement: Supplementary file 1 — Table S1 The primers of three loci located on IGF1. [file BRB3-14-e3432-s001.docx]

**Supplementary**

Table 1 the primers of three loci located on IGF1

| **Loci** | **Forward primer** | **Reverse primer** | **Single nucleotide extension primer** |
| --- | --- | --- | --- |
| rs5742612 | ACGTTGGATGCCTCCATAGGTTCTAGGAAA | ACGTTGGATGAGATTGGAAGACAGCACTCG | agggTCTAGGAAATGAGATCACACC |
| rs6218 | ACGTTGGATGGCAGAAGACTGCCTATAAAG | ACGTTGGATGTCTTCCCAAGATGGCACTTC | tgagcGGGAATAATTTTAAAAGGTACAC |
| rs6214 | ACGTTGGATGGTCCCCAGTGTGTACCTTTT | ACGTTGGATGTCACATCTAACTATGACAG | atagtCAGTCTTCTGCAGACTTAAC |

Abbreviations: IGF, insulin growth factor
